# 3D-Printed Plasmonic Nanocomposites: VAT Photopolymerization for Photothermal-Controlled Drug Release

**DOI:** 10.3390/ph17111453

**Published:** 2024-10-30

**Authors:** Ignacia Paz Torres Fredes, Elizabeth Nicole Cortés-Adasme, Bruno Andrés Barrientos, Juan Pablo Real, Cesar Gerardo Gomez, Santiago Daniel Palma, Marcelo Javier Kogan, Daniel Andrés Real

**Affiliations:** 1 Department of Pharmacological and Toxicological Chemistry, University of Chile, Santos Dumont 964, Santiago 8380494, Chile; ignacia.torres@ug.uchile.cl (I.P.T.F.); elizabeth.cortes@ug.uchile.cl (E.N.C.-A.); 2 Advanced Center of Chronic Diseases (ACCDiS), Universidad de Chile, Santos Dumont 964, IndePendencia, Santiago 8380494, Chile; 3 Unidad de Investigación y Desarrollo en Tecnología Farmacéutica (UNITEFA-CONICET), Haya de la Torre y Medina Allende, Córdoba X5000XHUA, Argentina; brunoabarrientos@gmail.com (B.A.B.); juan.real@unc.edu.ar (J.P.R.); sdpalma@unc.edu.ar (S.D.P.); 4 Departamento de Ciencias Farmacéuticas, Facultad de Ciencias Químicas, Universidad Nacional de Córdoba, Haya de la Torre y Medina Allende, Córdoba X5000XHUA, Argentina; 5 Departamento de Química Orgánica, Facultad de Ciencias Químicas, Universidad Nacional de Córdoba, Córdoba X5000XHUA, Argentina; cesar.gomez@unc.edu.ar; 6 Instituto de Investigación y Desarrollo en Ingeniería de Procesos y Química Aplicada (IPQA), Consejo Nacional de Investigaciones Científicas y Técnicas (CONICET), Haya de la Torre y Medina Allende, Córdoba X5000XHUA, Argentina

**Keywords:** 3D printing, controlled drug release, photothermal drug delivery, niclosamide, vat photopolymerization

## Abstract

Background: Gold nanoparticles can generate heat upon exposure to radiation due to their plasmonic properties, which depend on particle size and shape. This enables precise control over the release of active substances from polymeric pharmaceutical formulations, minimizing side effects and premature release. The technology of 3D printing, especially vat photopolymerization, is valuable for integrating nanoparticles into complex formulations. Method: This study aimed to incorporate gold nanospheres (AuNSs) and nanorods (AuNRs) into polymeric matrices using vat photopolymerization, allowing for controlled drug release with exposure to 532 nm and 1064 nm wavelengths. Results: The AuNSs (27 nm) responded to 532 nm and the NRs (60 nm length, 10 nm width) responded to 1064 nm. Niclosamide was used as the drug model. Ternary blends of Polyethylene Glycol Diacrylate 250 (PEGDA 250), Polyethylene Glycol 400 (PEG 400), and water were optimized using DesignExpert 11 software for controlled drug release upon specific wavelength exposure. Three matrices, selected based on solubility and printability, underwent rigorous characterization. Two materials achieved controlled drug release with specific wavelengths. Bilayer devices combining AuNSs and AuNRs demonstrated selective drug release based on irradiation wavelength. Conclusions: A pharmaceutical device was developed, capable of controlling drug release upon irradiation, with potential applications in treatments requiring delayed administration.

## 1. Introduction

For a drug to effectively produce its therapeutic effect, it must interact at its designated site of action, inducing a response. The primary goal of a pharmaceutical formulation is to ensure the safe and efficient administration of the drug, achieving plasma concentrations that stay within therapeutic ranges and avoid toxic levels [1].

The challenge with conventional pharmaceutical delivery systems lies in the idea that a drug disperses throughout the entire body rather than exclusively targeting the location where its therapeutic effects are needed. This becomes problematic when a drug affects organs or tissues beyond the intended target. Consequently, to ensure that the required amount of a drug reaches the target site, high and repetitive doses must be administered, leading to drawbacks such as the emergence of adverse effects, increased susceptibility to toxicity, and compromised quality of life for the patient [2].

The concept of guiding drugs to a desired action site emerged to address the abovementioned issues. This led to various strategies for the administration routes and pharmaceutical formulations through which active molecules are dispensed. A pharmaceutical formulation determines biopharmaceutical parameters, including release mechanisms, consequently influencing drug bioavailability and efficacy [3,4]. Modified release formulations permit the manipulation of release kinetics and, consequently, the duration that a drug remains in the body. Furthermore, formulations can be tailored to release a drug at specific spatial–temporal sites to facilitate a molecule’s interaction at the pharmacological action site [5].

One of the most groundbreaking strategies used for molecule targeting or vectorization involves the utilization of nanotechnology. The reduction of particle sizes to nanoscale dimensions offers several advantages, such as an increased surface-to-volume ratio, which facilitates the functionalization of nanoparticles [6,7]. This enables the attachment of molecules to their surface, thus facilitating targeted delivery. Additionally, due to their reduced size, the dissolution rate of poorly soluble drugs improves, enhancing their bioavailability and permeation through biological membranes [8].

Various materials are available within nanoparticle synthesis, encompassing both organic and inorganic components. Organic nanostructures include nanomicelles, nanoliposomes, nanocapsules, dendrimers, and polymeric nanoparticles [9]. Inorganic materials encompass metals like copper, silver, iron, silica, and gold. These materials can adopt various shapes and sizes, such as spheres, rods, prisms, stars, etc. Moreover, metallic nanomaterials possess optical and magnetic attributes that arise from the interaction of nanoparticles with light [10]. Gold, in particular, stands out due to its biocompatibility, low cytotoxicity, robust stability owing to gold–sulfur bonding, and straightforward synthesis. All these attributes position gold nanoparticles as promising candidates for biological applications [11].

**Figure 1:**
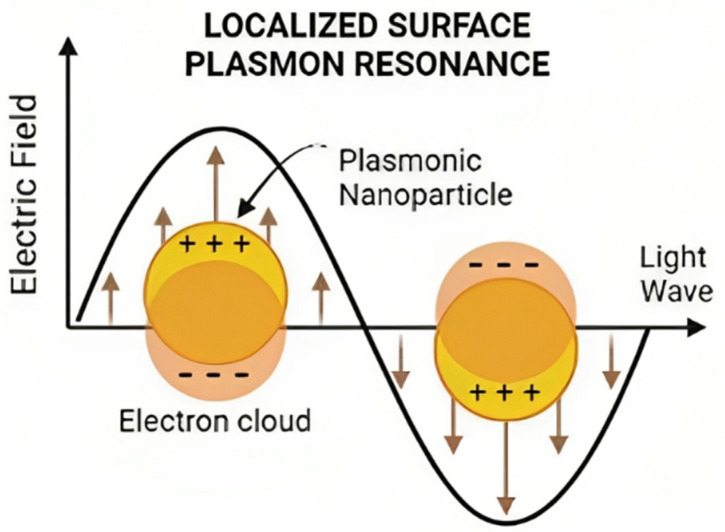
Schematic representation of Localized Surface Plasmon Resonance.

Gold nanoparticles (AuNPs) exhibit optical and magnetic traits attributed to their substantial electron density on the surface conduction band, facilitating the free movement of electrons. Localized Surface Plasmon Resonance (LSPR) pertains to the synchronized oscillation of electrons in the outermost atomic layer of a nanostructure’s surface [12]. The oscillation and excitation stem from the interaction between the nanoparticle and light, as depicted in Figure 1. This interaction culminates in heightened local energy dissipation, resulting in photothermal effects in the context of gold nanoparticles [13].

The size and shape of the nanoparticle will impact the energy required to trigger LSPR [14]. This outcome is due to the restrictions placed on electron movement by the system’s size and shape. When more electrons are present in the nanostructure, such as in nanorods with larger surface areas, the signal’s dispersal widens, demanding less energy to interact with the sample and excite these electrons [15]. Consequently, larger nanoparticles necessitate less energy to stimulate electrons, leading to longer wavelengths due to their inverse proportionality. This explains why nanorods’ plasmon occurs near 1064 nm, whereas smaller nanospheres with reduced surface areas exhibit plasmons close to 520 nm. These plasmonic nanoparticle properties prove invaluable for drug targeting, controlled release, and photothermal therapy [16].

However, a significant hurdle in nanoparticle application for pharmaceutical formulations is their integration into solid forms without compromising their distinct attributes. A pioneering solution to this challenge involves 3D printing. This additive manufacturing technique crafts three-dimensional solid objects guided by digital instructions [17,18].

In essence, 3D printing permits the creation of solid structures with varied sizes and shapes, achieving precise customization. It allows for the amalgamation of materials possessing diverse physicochemical properties and inks laden with various active agents or loaded nanomaterials [19]. These can be strategically positioned in different print layers or surfaces without mixing. This technology facilitates the realization of innovative geometries that elude traditional methodologies, like hollow or porous structures.

Within 3D-printing techniques, VAT photopolymerization (VATp) shines. It encompasses a suite of methods employing liquid resins that cure and solidify under light-induced photopolymerization. The prevalent VATp process entails pouring liquid resin into a reservoir. As printing commences, the print bed descends into the resin, leaving a thin fluid layer between the bed and the tank bottom. A UV laser traces a cross-sectional pattern of the 3D model while solidifying the material. This unfolds layer by layer. Upon completing a layer, the bed rises, allowing the liquid resin to flow and prepare for the subsequent layer’s printing [20].

Materials include photosensitive polymers like Polyethylene Glycol Diacrylate (PEGDA) of varying molecular weights, polyethylene glycol (PEG), and photoinitiators such as phosphine oxides [21,22]. VATp’s advantages over other techniques encompass superior print resolution and precision. It boasts versatility by designing various photosensitive liquid resins capable of incorporating diverse molecules like drugs and nanoparticles while in solution. Additionally, this technique boasts rapid execution at room temperature.

Combining plasmonic nanoparticles and 3D printing leverages the outlined benefits, fostering synergy between precision in creating three-dimensional constructs, customization, and the fusion of different materials in a 3D-printed device alongside plasmonic nanoparticles’ optical and magnetic attributes.

Responsiveness to stimuli is paramount to govern the drug release kinetics from a matrix or 3D-printed pharmaceutical device. Internal stimuli encompass pH fluctuations, ion presence, and enzymatic activity, though these variables often hinge on individual patient circumstances [23]. External stimuli, like light, remain unaffected by patient physiological conditions. Thus, this study evaluates the integration of gold nanospheres and nanorods into polymeric matrices via VATp 3D printing.

In this way, the aim was to design the formulation of a polymeric-based pharmaceutical platform capable of controlling the release of an active ingredient through irradiation with wavelengths proximal to the near-infrared (NIR) plasmon. Among the potential applications of this project is the ability to generate multiple-dose devices that can be used in the treatment of chronic diseases such as cancer, where early exposure doses and maintenance doses are needed. It can also be applied to treatments where administering two different active ingredients is required, such as in post-operative care, where a dose of analgesia and another of antibiotics could be released from the same device with a single application, subjected to irradiation. The proposed platform was designed based on a model drug’s solubility and potential administration. Niclosamide (NICLO), derived from salicylic acid, is a class II drug under the Biopharmaceutical Classification System (BCS), marked by low solubility and high permeability. Initially an anthelmintic for parasites like *Taenia* spp., the drug impedes oxidative phosphorylation in parasites. Presently, Niclosamide is being repurposed, showing efficacy against gastric, prostate, and breast cancers. It has also displayed promise against COVID-19 and Helicobacter pylori [24]. In the end, the formulation design was carried out based on the solubility of the model drug and its potential release. This design can thus be projected toward administering other medications of different classes according to the BCS.

Hence, this study endeavored to design, create, and characterize 3D-printed devices for photothermal therapy. It amalgamated VATp with gold nanoparticles for the controlled release of the model drug, based on bilayer devices combining nanospheres and nanorods, demonstrating their ability to selectively and precisely release the drug based on the irradiation wavelength.

## 2. Results and Discussion

### 2.1. Synthesis Process of Gold Nanoparticles with Different Morphologies

**Figure 2:**
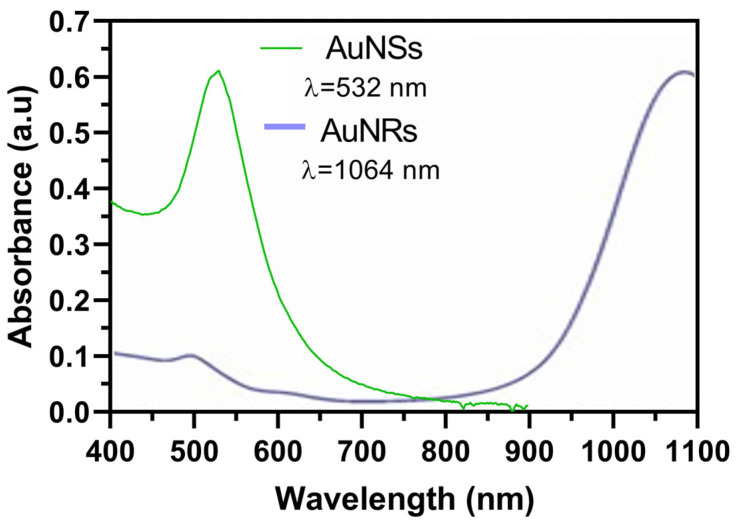
Plasmon resonance peaks of synthesized AuNSs and AuNRs determined by UV–Vis spectrophotometry.

In the initial phase of our research, we synthesized two distinct nanosystems characterized by their separate plasmonic bands. AuNSs and AuNRs were synthesized as a preliminary demonstration. Figure 2 illustrates the distinct plasmon resonance peak of AuNSs at 532 nm, and the two distinctive plasmon resonance peaks of AuNRs, the first one at 1064 nm, attributed to the longitudinal plasmon, and a second one at 530, which corresponded to the transversal one [16,25,26].

Table 1 provides a comprehensive insight into the characterization of the nanosystems by presenting data on Zeta potential and hydrodynamic diameter. This analysis serves as a means to derive essential parameters regarding colloidal stability, with Zeta potential values approaching ±30 mV indicating a state of stability. Such values signify the presence of an adequate electrostatic repulsion force, thus mitigating the risks of particle agglomeration and the ensuing destabilization of the colloidal suspension [16,26]. Moreover, determining the hydrodynamic diameter offers an approximation of particle size. However, it is essential to note that these values may be subject to the influence of the inherent anisotropy of each nanosystem and the measurement apparatus’s methodology, which relies on observing Brownian motion within the particle suspension.

In the case of AuNSs, the observed Zeta potential was −48 ± 3mV, reflecting their favorable colloidal stability. This negative potential was attributed to the charge carried by the sodium citrate employed in synthesizing AuNSs [16]. Additionally, the hydrodynamic diameter measurement yielded a value of 27 ± 1.2 nm. It is worth noting that while the anticipated value, as per the synthesis protocol, hovered around 10 nm, the hydrodynamic diameter measurement encompassed the solvation sphere, thus allowing for a slight increment in the measurement value.

For the AuNRs, the Z potential exhibited a value of 32 ± 0.3 mV, indicating the desired colloidal stability. This positive value can be attributed to the presence of the CTAB stabilizer used in their synthesis, which contains an ammonium group in its structure, imparting a positive charge [26]. In addition, the transversal hydrodynamic diameter measured 10 ± 1 nm, while the longitudinal diameter measured 60 ± 16 nm. However, it is essential to note that due to the anisotropic nature of these structures, the obtained values may be subject to alteration, and the values considered for analysis were those obtained through electron microscopy.

### 2.2. Characterization of Nanosystems by NTA and STEM

**Figure 3:**
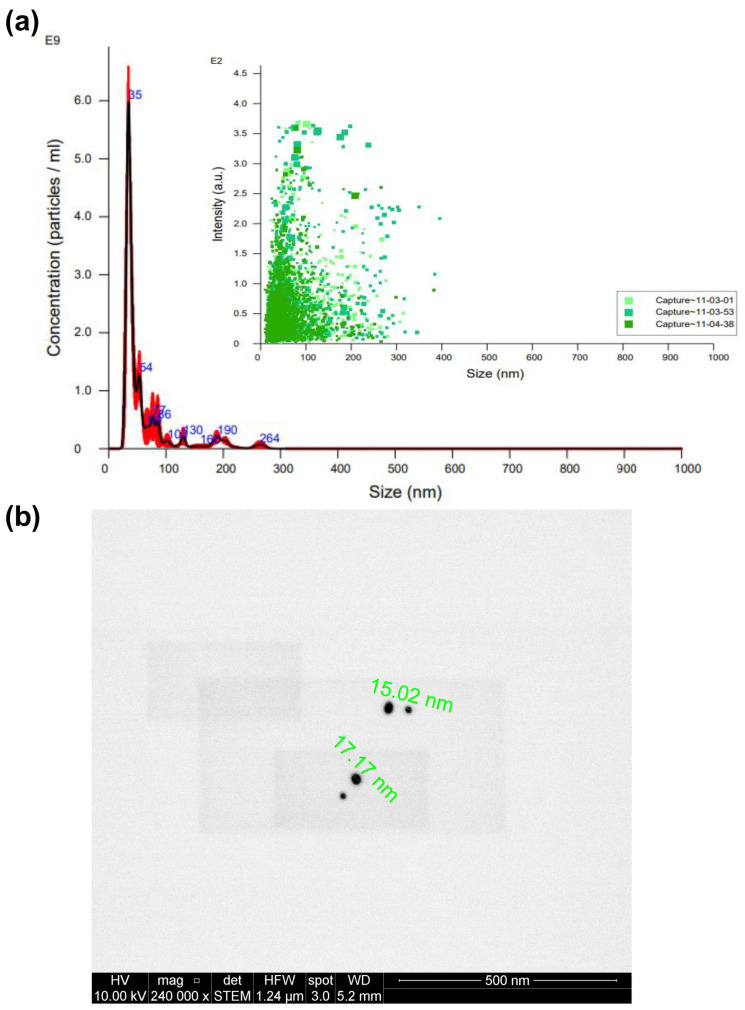
Characterization of AuNSs by (**a**) NTA (different captures of the same sample obtained are represented in different green tones) and (**b**) STEM.

Figure 3a presents an examination of the nanospheres’ average size, approximately 35 nm, as determined through NTA. Additionally, this figure illustrates a broader distribution of particles ranging from 0 to 100 nm, with an average nanoparticle concentration of 176 pM. In Figure 3b, we observe the nanospheres’ morphology, as captured through STEM, revealing an average diameter of approximately 16 nm.

Notably, the size results for AuNSs derived from NTA appear more prominent than those obtained through STEM. This discrepancy could have arisen from the potential presence of nanoparticle aggregates in the solution, which NTA may have detected [13]. However, electron microscopy directly visualizes individual nanoparticles, rendering the values acquired via STEM the preferred reference. Consequently, we can confidently conclude that the synthesis of nanospheres yielded the desired size and shape.

**Figure 4:**
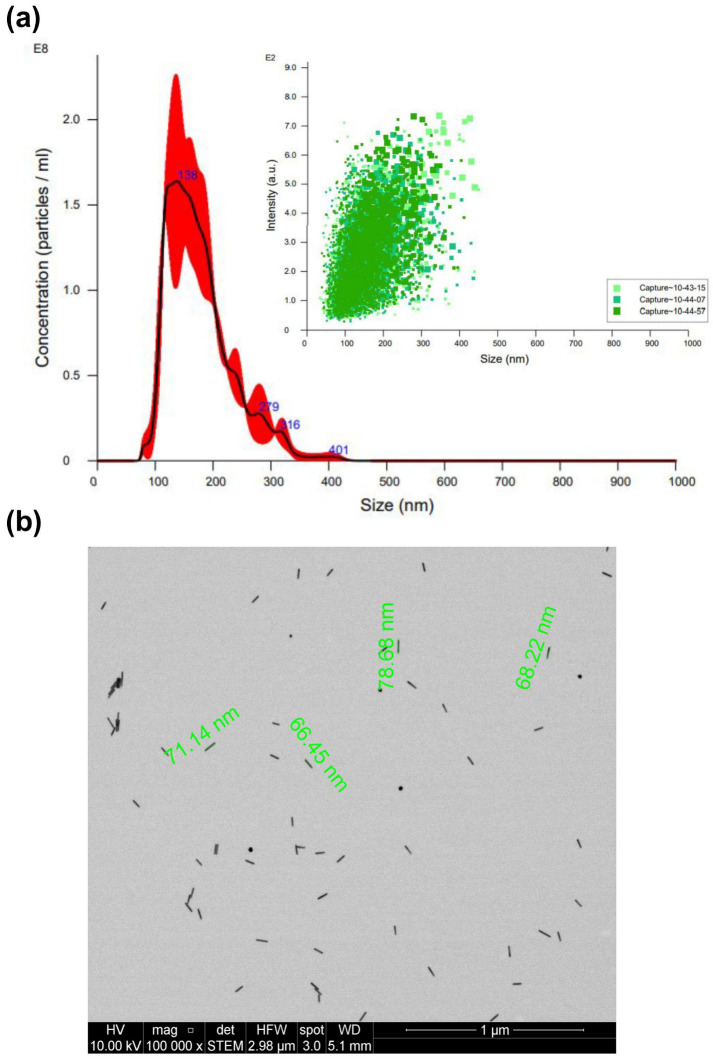
Characterization of AuNRs by (**a**) NTA (different captures of the same sample obtained are represented in different green tones) and (**b**) STEM.

Figure 4a presents the average size of AuNRs, measuring 138 nm, along with a broader particle distribution spanning the 100 to 200 nm range. The average nanoparticle concentration after synthesis was 26.6 pM. Subsequently, Figure 4b illustrates the morphological characteristics of AuNRs through STEM, yielding an estimated average diameter of approximately 71 nm. Notably, the size value obtained from STEM is less than that derived from NTA. This variance can also be attributed to the inherent anisotropic nature of AuNRs [25,26].

### 2.3. Evaluation of the Solubility of Niclosamide in Ternary Mixtures

Utilizing a mixed-centered design approach and employing the Design Expert software, we successfully formulated ternary mixtures comprising PEGDA 250, PEG 400, and water (used as the suspension medium for the nanosystems), as detailed in Table 2. This methodology enabled an examination of the solubility of the model drug, Niclosamide, within each of the proposed matrices.

The photosensitive polymer, PEGDA, plays a pivotal role in controlling the extent of crosslinking among the monomers during the solidifying of the liquid resin. This crosslinking degree significantly influences the resulting rheological properties and the ultimate print conditions [20]. Additionally, it is essential to note that within all matrices, a minimum concentration of 10% of water was consistently present, as it served as the vehicle for incorporating the nanosystems.

**Figure 5:**
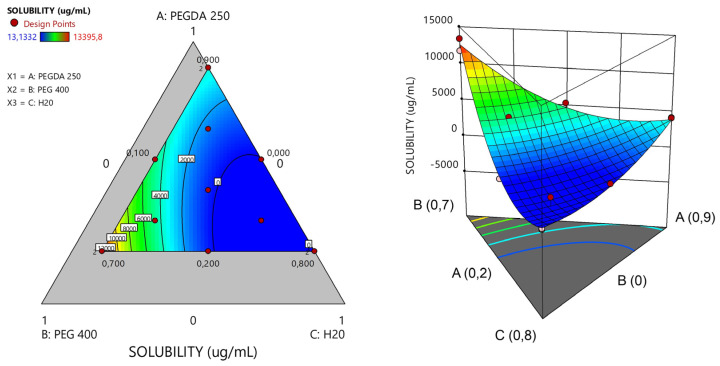
Response surface plots of ternary PEGDA/PEG400/water mixtures. Evaluation of the solubility of the model drug Niclosamide at 25 °C.

In the study of the 13 matrices, a response surface graph was generated, as depicted in Figure 5. It became apparent that matrices with an escalating proportion of PEG 400 exhibited higher solubility of Niclosamide, followed by PEGDA. NICLO may be more soluble in PEG 400 than in PEGDA 250 due to the structural and chemical differences between these two compounds. On the one hand, PEG 400 is a linear polymer of ethylene oxide with a flexible chain and numerous hydrophilic groups (-OH and -O-). This structure allows for more hydrogen bonding interactions with water molecules and hydrophilic drugs, thereby enhancing solubility. In contrast, PEGDA 250 also contains polyethylene glycol chains but is functionalized at both ends with acrylate groups, which tend to be more reactive and less hydrophilic than the terminal groups in PEG. The presence of the acrylate groups reduces the flexibility of the molecule and may limit hydrogen bonding interactions with the drug. Additionally, PEG 400 has lower viscosity compared to PEGDA 250. The higher viscosity of PEGDA 250 could limit the mobility of drug molecules within the solvent, thereby decreasing solubility. In summary, PEG 400 is more flexible, more hydrophilic, and has a less reactive structure than PEGDA 250, which may increase a drug’s solubility in PEG 400 due to its greater ability to interact with the drug at the molecular level [20].

To proceed with the characterizations, matrices capable of solubilizing the highest concentration of Niclosamide were selected to ensure maximum drug loading. It is important to emphasize that the drug exhibited greater solubility in the non-photopolymerizable polymer (PEG 400). Consequently, matrices with the highest solubility of NICLO were chosen, featuring varying ratios between the non-photopolymerizable polymer and PEGDA 250. Specifically, three matrices with different percentages of PEGDA and varying PEGDA/PEG 400 ratios—labeled runs 2, 5, and 6 (highlighted in bold)—were selected for further evaluation of their printing capabilities.

Table 3 presents the proportions of PEGDA, PEG 400, and water in these selected matrices, along with the Niclosamide drug loading in μg/mL for each matrix. Matrix 6 had the highest PEGDA 250 content at 90%, followed by Matrix 5 at 55% and Matrix 2 at 20%. All selected matrices contained 10% water (with the nanosystems suspended), and the remaining volume was filled with the cosolvent PEG 400. The concentrations of AuNSs and AuNRs used were 17.6 pM and 2.66 pM, respectively, in a total volume of 50 mL per matrix. Lower concentrations of AuNRs were chosen due to their higher photothermal efficiency compared to AuNSs.

### 2.4. Rheology

To further characterize the matrices, a rotational rheological test was conducted to understand the viscoelastic behavior of the materials before the photopolymerization process.

From a rheological point of view, stereolithography (SLA) resins are predominantly subjected to Couette flow, so it is most relevant to perform rotational rheometry studies [27]. One of the aspects to consider is that the impressions are created upside down, so a low shear viscosity is always necessary to allow the formulation flow on the moving platform. However, at the same time, once a part of the structure is formed, good mechanical properties and the ability to resist low stresses are required to prevent the object from detaching from the platform and tolerate successive layers.

**Figure 6:**
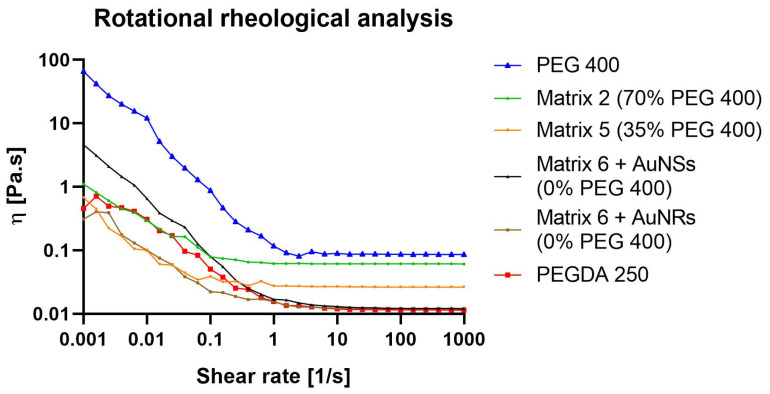
Rotational rheological analysis of PEGDA 250, PEG 400, and 3D-printing matrices 2, 5, and 6 (with AuNSs or AuNRs).

For SLA printing, both the operating viscosity and shear rates are considerably small. In general, the viscosity of the photopolymer should be less than 5 Pa.s at 30 s^−1^, which ensures that the photopolymer flows freely and can form a new layer ready for polymerization [28]. This viscosity value will ultimately depend on the printer and the settings used. As depicted in Figure 6, PEGDA 250 exhibited the lowest viscosity as the shear rate increased, while, conversely, PEG 400 displayed the highest viscosity. This behavior may be attributed to the higher molecular weight of PEG 400, which promotes a more pronounced material structuring. On the other hand, Matrix 6 exhibited a similar behavior to PEGDA 250, likely due to it being the major component in the mixture. In this case, the influence of AuNSs and AuNRs was evaluated, and it was observed that at lower shear rates, systems with AuNRs had viscosities more closely resembling those of PEGDA 250. This could be attributed to CTAB traces resulting from synthesizing these nanosystems. The surfactant behavior of this compound may ensure better miscibility between water and the photopolymer, contributing to the viscoelastic behavior of the system resembling the latter, which is the major component. Matrices 5 and 2 exhibited higher viscosities, with a trend correlating with the incorporation of PEG 400, with Matrix 2 being more viscous, containing 70% of PEG 400.

### 2.5. 3D Printing

Matrices denoted as run 2, 5, and 6 were proposed to be fabricated, utilizing PEGDA 250 as the printing material. The intended design for the printing involved creating cylinders with 3 × 5 mm dimensions. This design was developed using Fusion 360 software and subsequently prepared for printing through slicing in Chitubox 64 software, resulting in 100 layers. The digital file was then transmitted to the Anycubic Photon S printer (Anycubic Technology Co., Shenzen, China), commencing the printing process, which lasted approximately 20 min. Upon completion of the printing procedure, the devices were washed and cured in the Anycubic Wash and Cure station for 1 min. The resulting devices, post-printing, underwent characterization through optical microscopy, weight uniformity assessment, and SEM examination.

### 2.6. Images Obtained by Optical Microscopy

**Figure 7:**
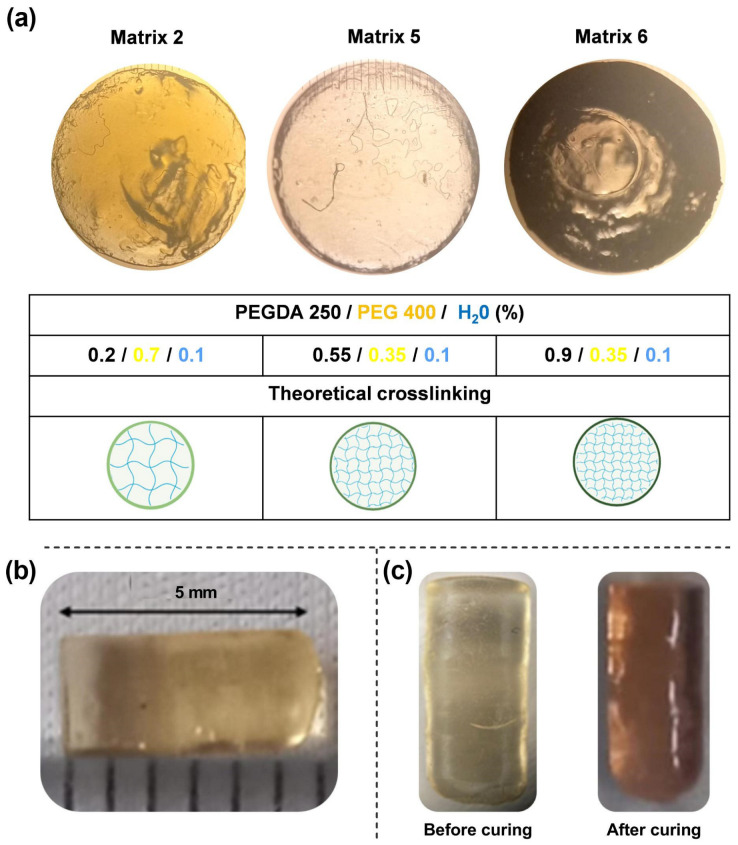
(**a**) Microphotographs of prints acquired through optical microscopy. (**b**) Images of the device resulting from Matrix 6 with PEGDA 250. (**c**) Device before and after the curing process.

Matrices 2, 5, and 6 were formulated for printing, resulting in the solid structures depicted in Figure 7a, as observed through optical microscopy. The solid structures in Matrices 2 and 5 did not conform to the anticipated dimensions. Nevertheless, these deviations were instrumental in conducting a preliminary investigation to identify the operational parameters, wherein the proportions of the matrix constituents would enable successful printing. Conversely, Matrix 6 exhibited diminished light transmission, suggesting a potentially greater extent of polymer crosslinking (as indicated in Figure 7c). This observed phenomenon could be attributed to the escalating concentrations of PEGDA 250.

The correlation between the computer-designed geometry and the printed systems varied depending on the printing material. In all cases, the cross-sectional area of the prints was statistically consistent, with an average width of 2.87 ± 0.1 mm. However, the systems printed with Matrices 2 and 5 did not reach the expected length of 5 mm, resulting in smaller prints with approximate lengths of 2 mm. Figure 7b shows a print obtained with Matrix 6. In the case of the objects printed with this matrix, the correlation was accurate concerning the digital file. Based on these results, it can be concluded that the presence of PEGDA 250 is crucial for ensuring suitable printability parameters.

Furthermore, the printed objects changed color after curing, as seen in Figure 7c. This could be attributed to the higher power of the post-curing lamp, which resulted in more crosslinking, changing the material’s structure. This effect could have arisen for two main reasons. First, during the curing process, the degree of polymerization of PEGDA increased, which resulted in a color change [29]. Second, this effect, combined with the presence of gold nanoparticles, altered the color of the material due to changes in the environment where they were located. It is noteworthy that this effect was observed in all matrices. Therefore, it can be concluded that the effect is more related to the presence of gold nanoparticles, as the photopolymerization of the materials was likely different due to changes in the PEGDA/PEG ratios in the selected matrices. Other authors have reported similar findings. In a specific study, it was demonstrated that when higher concentrations of gold chloride solutions were used, the absorption peak of the materials shifted from approximately 540 nm to 560 nm, resulting in a color change of a hydrogel from a lighter to a darker tone, such as bluish-purple [30].

### 2.7. FTIR

**Figure 8:**
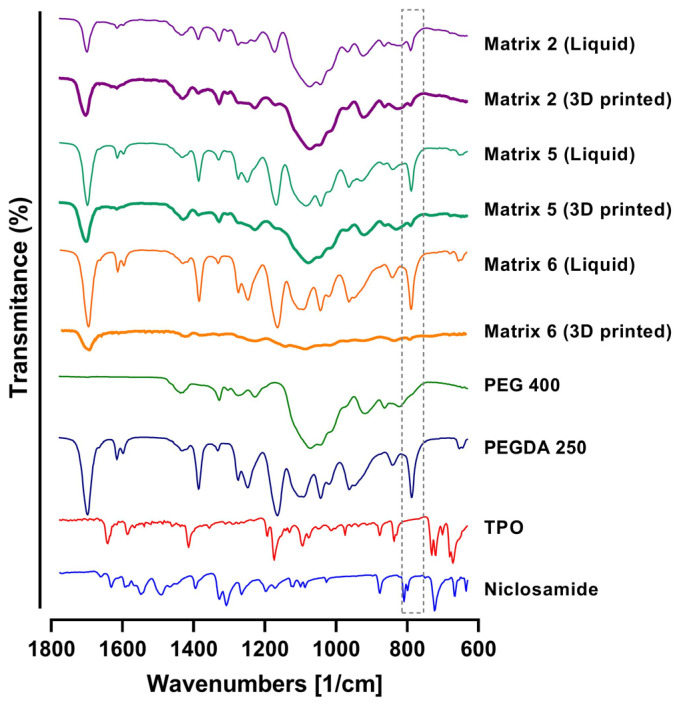
FTIR spectra of PEGDA, PEG 400, TPO, Niclosamide, and 3D-printing Matrices 2, 5, and 6. Note: the gray dotted box is to focus in the decrease in the signal of the C=C bonds in the 3D printed matrixes.

To assess the photopolymerization process and potential interactions between the components, the FTIR spectra of the matrices were analyzed both in their nonphotopolymerized state (liquid materials) and following the photopolymerization process (3D-printed materials). In the analysis, the characteristic bands of PEGDA were attributed to the C-O bonds of the ester groups (at 1270 cm^−1^, 1190 cm^−1^, and 985 cm^−1^) [31,32]. Following the curing process, the FTIR spectra of the matrices exhibited significant changes due to irradiation and copolymerization. Indeed, under UV irradiation, the photoinitiator generated active radicals that opened the double bonds in the monomers, thereby promoting crosslinking reactions. Photopolymerization was confirmed by monitoring double bond peaks at 1636, 1618, 1410, and 810 cm^−1^ [33,34]. As depicted in Figure 8, by the end of the UV curing step, in the case of the PEGDA sample, the double bond peaks were markedly reduced, signifying their continuous combination with the generated radicals, contributing to curing and augmenting crosslinking density3. The absence of infrared bands assignable to unsaturated C=C bonds (highlighting the 810 cm^−1^ band) in the 3D-printed material spectra suggested the successful curing of the formulations. This outcome is particularly pertinent to biomedical applications, as the conversion of double bonds is intricately linked to material biocompatibility. The presence of unreacted free radical groups can potentially lead to irritation and damage to soft tissue [35].

### 2.8. Weight Uniformity

The uniformity of weight of the printed devices was studied to obtain a parameter for print quality. This allowed for the evaluation of the printing accuracy and the matrices’ printability [24]. Additionally, the drug capacity of each print was estimated based on its weight and the initial amount of Niclosamide dissolved in the matrix.

Ten prints were weighed on an analytical balance for uniformity, and their average weight and standard deviation were calculated. Table 4 shows the average weight of 10 prints for all printed matrices. For Matrix 2, the average weight was 1.97 mg with a standard deviation of 0.34. This indicated that the print quality for this matrix was unfavorable. However, the standard deviation among the ten samples was less than 0.4. This suggests that although the printing did not achieve the expected outcome, the printed devices were consistent. In other words, the print was not exact but precise. Additionally, it is worth noting that the estimated amount of Niclosamide, while approximately 0.019, may not have been accurate due to irregularities in the printing.

**Figure 9:**
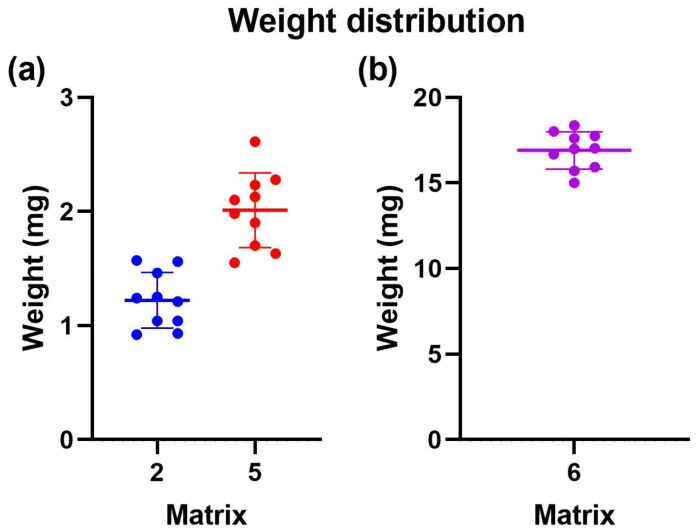
Weight uniformity of 3D-printed devices employing different photopolymerizable matrixes. (**a**) Distribution of weight uniformity for matrix 2 in blue and matrix 5 in red. (**b**) Distribution of weight uniformity for matrix 6 in violet.

Like Matrix 2, Matrix 5 included prints where the devices remained stuck at the bottom of the tank rather than on the print bed, presenting irregularities (Figure 9a).

On the other hand, for Matrix 6, the average weight was 14.97 mg, with a standard deviation of 0.1 (Figure 9b). Only some devices remained stuck in the tank, while others adhered to the print bed. From this, it can be concluded that Matrix 6, which contained more PEGDA 250, had the most significant potential for 3D printing using VAT photopolymerization. Due to the above information, only Matrix 6 was selected for the subsequent tests.

### 2.9. SEM

**Figure 10:**
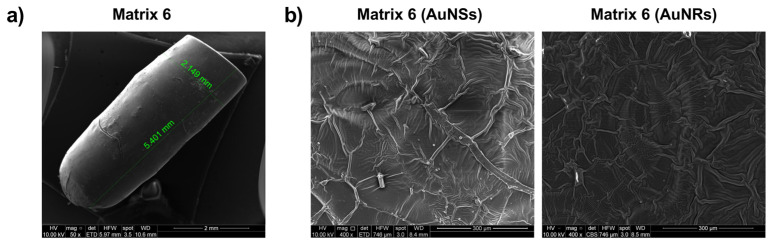
Characterization of prints obtained with Matrix 6 loaded with AuNSs and AuNRs using SEM.

The prints from Matrix 6 containing AuNSs and AuNRs were visualized using SEM to assess their surface structure. As depicted in Figure 10a, the generated prints exhibited a structure similar to the digital file, with a smooth surface and no noticeable difference between the printing layers. In Figure 10b, the surfaces of the prints with AuNSs (left) and AuNRs (right) are shown in detail. In both cases, the presence of drug crystals was not observed, and the nanosystems on the surface were not prominent.

**Figure 11:**
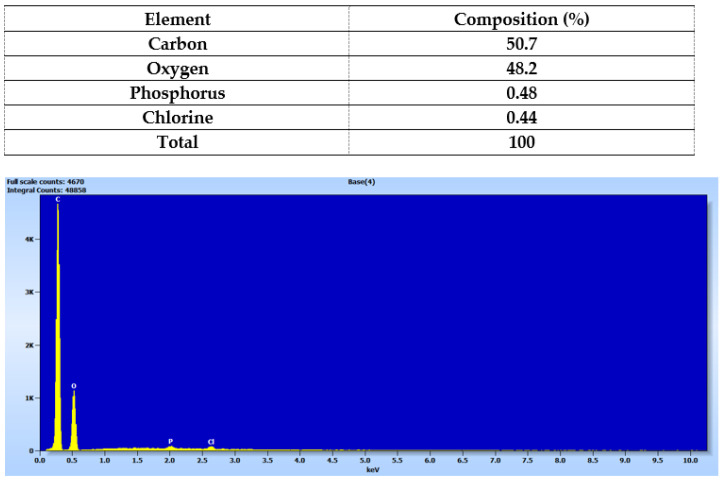
Elemental analysis of the solid device obtained using Matrix 6.

Elemental analysis of the printed device from Matrix 6 with PEGDA 250 and AuNSs was conducted using Energy Dispersive X-ray Spectroscopy (EDX), as shown in Figure 11, where the percentage composition data for each element are also presented [19,24]. Carbon and oxygen were the most abundant elements in the solid, attributable to the organic molecules forming part of its structure, such as PEGDA 250 and PEG 400. The presence of chlorine can be attributed to the niclosamide molecule, confirming the successful loading of the drug into the device. The presence of phosphorus may have been due to the TPO molecule, which contains this element in its structure.

According to the photopolymerization reaction, the TPO molecule degrades into two free radicals, benzoyl and phosphorus acid, which initiate the polymerization reaction of PEGDA 26 by providing these free radicals to the available acrylate ends [34]. Therefore, in the future, it would be essential to characterize the form in which this phosphorus is present, whether as a radical or as an unreacted TPO molecule.

It is important to note that the elemental analysis did not reveal the presence of gold. This suggests that the nanoparticle concentrations used may have been too small to be detected by the equipment or that they were better distributed within the core of the prints rather than on the surface.

### 2.10. Photothermal Effect of the Prints

**Figure 12:**
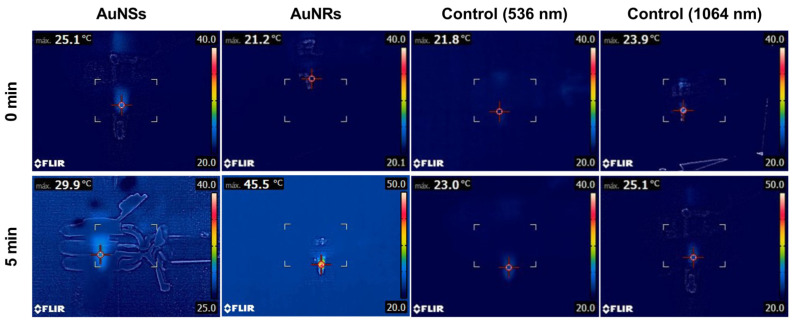
The photothermal effect of controls and the printed device from Matrix 6 with AuNSs or AuNRs after 5 min of irradiation with a 536 nm or 1064 laser, respectively.

To evaluate the inclusion of nanoparticles in the prints, the photothermal effect was assessed by irradiation with a 532 nm laser for the prints containing AuNSs and 1064 nm for the prints with AuNRs [26]. As a control, prints were generated without nanoparticles, and temperature gradients of approximately 1 °C were observed after 5 min of irradiation with both lasers (Figure 12). The photothermal effect for the print of Matrix 6 with AuNSs showed an increase in temperature from 25 °C to 29.9 °C after 5 min of irradiation with the 532 nm laser. The fact that the device increased the temperature by approximately 5 °C after irradiation indicates that the nanoparticles were correctly incorporated into the matrix and that the concentration was sufficient to produce a photothermal effect. The same was verified in the case of systems with AuNRs, where a change in temperature from 21.2 °C to 45.5 °C was observed after 5 min of irradiation. In this case, the gradient was approximately 24.3 °C at the same time, despite the nanoparticle concentration being ten times lower. This confirms the superior photothermal efficiency of AuNRs compared to AuNSs [26].

### 2.11. DSC and TGA

**Figure 13:**
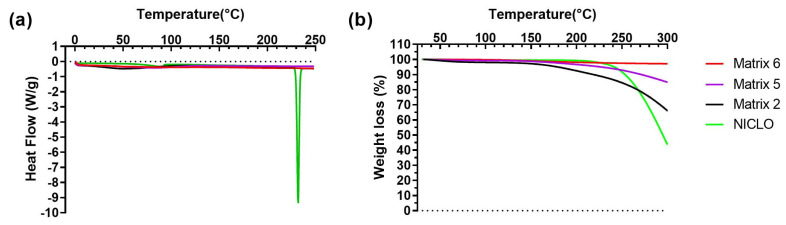
DSC (**a**) and TGA (**b**) analyses of Niclosamide and 3D-printed objects obtained using Matrices 2, 5, and 6.

Based on the demonstrated photothermal effect of the 3D prints, thermal tests were conducted on the materials. In Figure 13a, the results obtained through DSC are displayed. Niclosamide exhibited a characteristic melting peak at 233 °C, far from the temperature values reached during laser irradiation [24]. Therefore, no changes in its crystalline structure or potential degradation due to this process were expected. In the case of prints using all matrices, the drug’s characteristic peak was not observed, indicating a potential amorphization of the drug due to photopolymerization.

Figure 13b presents the results of TGA. As seen, Niclosamide experienced a decrease in mass after its melting point (233 °C). For prints from Matrices 2 and 5, no changes in mass were observed below 150 °C. Subsequently, there was a decrease in weight, likely due to the loss of water and the PEG 400 present in the prints, with the effect being more pronounced in the materials of Matrix 2. In the case of Matrix 6, no changes were observed within the studied temperature range (up to 300 °C).

### 2.12. Drug Release from the Matrices Without Irradiation

**Figure 14:**
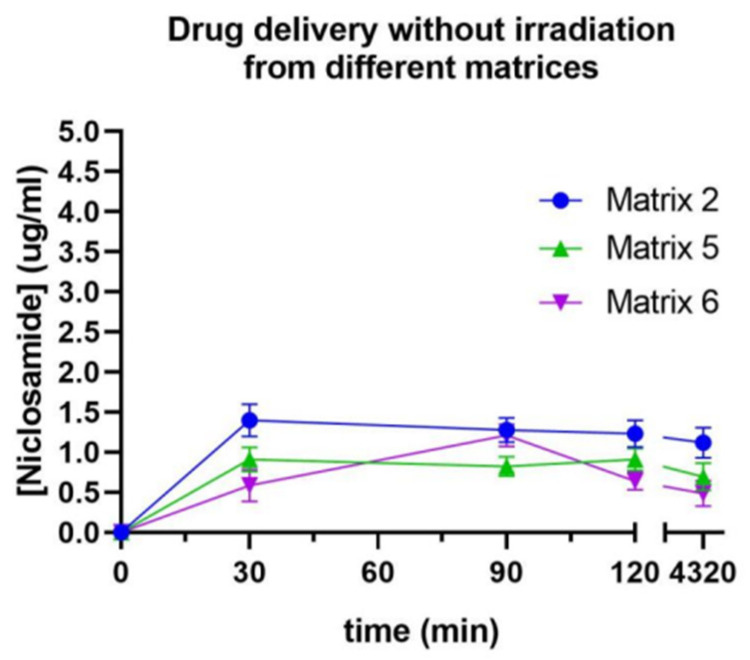
Release profile of Niclosamide without irradiation from Matrices 2, 5, and 6 measured by UV–Vis spectrophotometry.

The prints were subjected to release assays with and without irradiation to assess the selective drug release. Initially, dissolution tests were conducted to determine which of the evaluated materials prevented drug release without irradiation, as shown in Figure 14 [5]. In all cases, there was an initial release of the drug within the first 30 min, followed by a stable plateau that lasted for 4320 min (3 days). While no statistically significant differences were observed, the print that released the least amount of Niclosamide (0.5 µg/mL or 1%) was from Matrix 6, corresponding to its higher degree of crosslinking due to PEGDA 250.

Due to the poor release of Niclosamide when the device was not exposed to irradiation, Matrix 6 was selected for the subsequent release assays. The incorporation of nanosystems was expected to induce a change in the release of the active ingredient, either in the release rate or in the release mechanism. Therefore, this proportion of materials that allowed for the least amount of Niclosamide release when the devices were not subjected to irradiation was selected, with the expectation that this percentage would increase when the device was exposed to the stimulus.

### 2.13. Drug Release from Prints with AuNSs and AuNRs and the Bilayer Device Under Irradiation at Different Wavelengths

The concentration of Niclosamide released over time was evaluated following the irradiation of printed devices using Matrix 6 with either AuNSs or AuNRs and a bilayer device that combined both materials in different layers. For the assay, the printed devices were suspended in 1.5 mL of PBS, and samples were collected every 1 min of irradiation up to 15 min. The samples were measured using a UV–Vis spectrophotometer to estimate the Niclosamide concentration based on the previously constructed curve.

**Figure 15:**
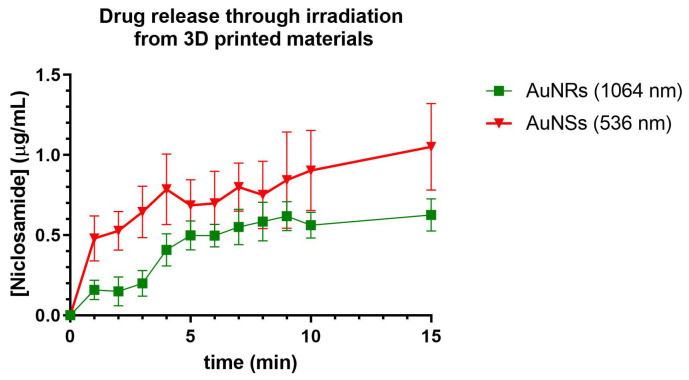
Release of Niclosamide from the print of Matrix 6 of AuNSs after irradiation with the 536 nm laser. Niclosamide released from the print of Matrix 6 with AuNRs following irradiation with the 1064 nm laser. Data obtained from the UV–Vis spectrophotometer.

In Figure 15, the release of Niclosamide from the print of Matrix 6 with AuNSs following irradiation with the 532 nm laser was depicted. A rapid increase in the Niclosamide concentration was observed in the first 10 min of irradiation (0.9 µg/mL, release rate 0.09 µg/min), followed by a decrease in the release rate up to minute 15 (1 µg/mL, release rate 0.02 µg/min), reaching 2% Niclosamide release after 15 min of irradiation.

Compared to those devices without irradiation, the irradiated Matrix 6 with AuNSs achieved the same percentage of Niclosamide release as the non-irradiated matrix in half the time (15 min). This could indicate that the device was responsive to irradiation and that the AuNSs in the device could exert a photothermal effect in such a way that it increased the release rate of the drug incorporated into the print [26].

Niclosamide release was also evaluated following irradiation with a 1064 nm laser from 3D-printed devices using Matrix 6 with AuNRs. The release profiles were similar to those obtained with AuNSs, with sustained release for up to 10 min (0.5 µg/mL, release rate 0.05 µg/min), followed by a decline in release up to min 15 (0.55 µg/mL, release rate 0.01 µg/min). This also indicates that the device could respond to the stimulus of irradiation by releasing Niclosamide from the print.

**Figure 16:**
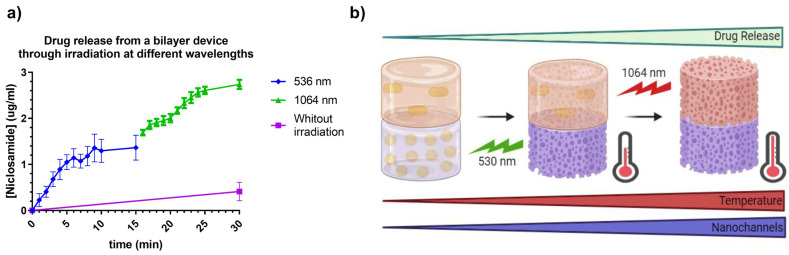
(**a**) Release of Niclosamide from the bilayer device after laser irradiation with 532 nm and 1064 nm. Data were obtained through measurement with the UV–Vis spectrophotometer. (**b**) Schematic representation of the designed photothermal-controlled drug release bilayer device.

It is important to note that the temperature reached by devices with AuNRs was higher, which would be expected to result in more outstanding drug release. However, the release rate was lower, possibly due to the lower concentration of nanoparticles within the 3D print. This parameter is significant as it could suggest that release may occur through the generation of nanochannels in the material, allowing the diffusion of the medium within the print, thereby facilitating drug release. In Figure 16, the release of Niclosamide from the bilayer device can be observed following continuous irradiation with the 532 nm and 1064 nm lasers, respectively. The irradiation methodology was the same as for the prints of Matrix 6 with AuNSs and AuNRsAfter this, irradiation with the 1064 nm laser was carried out, increasing the concentration of Niclosamide progressively until it reached a continuous concentration at 30 min. From these data, it can be concluded that the bilayer device could respond to irradiation at different wavelengths, releasing the model drug, Niclosamide, in a controlled manner.

As observed, the release during the first 15 min (irradiation at 532 nm) produced a profile similar to that obtained with the AuNSs print (Figure 15), with slightly higher values, showing a release of 1.2 µg/mL at 15 min. This increase of approximately 0.2 µg/mL could be attributed to the release from the AuNRs layer due to the small plasmonic band associated with the cross-sectional area of these nanoparticles and heat transfer between both layers. On the other hand, the release in the second 15 min was notably higher, reaching values of 2.8 µg/mL at 30 min. This increase could have been due to the device reaching higher temperatures due to the longer total irradiation time, as well as a possible increase in the number and dimensions of nanochannels produced in the material, increasing the swelling of the device, which could have led to an increase in drug release from both layers, as represented in Figure 16b.

## 3. Materials and Methods

### 3.1. Materials

NICLO (Lot 0000122971), Polyethylene glycol diacrylate 250 (PEGDA 250), Polyethylene glycol 400 (PEG 400), and diphenyl (2,4,6-trimethylbenzoyl) phosphine oxide (TPO) were procured from Sigma-Aldrich (St. Louis, MO, USA). Ultrapure Milli-Q water with a resistivity of 18.2 MΩ at 25 °C, filtered through a 0.22 µm pore size filter, was employed in this study. Unless otherwise specified, all other chemicals were of analytical grade.

### 3.2. Synthesis of Gold Nanoparticles of Different Morphologies

The methodology described by Petkova et al. was employed to produce gold NSs (AuNSs). The AuNSs were prepared through reduction using HAuCl_4_ citrate. An aqueous solution of HAuCl_4_ was brought to reflux and, after 10 min, an aqueous solution of sodium citrate (38.8 mM) heated between 50 and 60 °C was added. The system was refluxed in a round-bottom flask for 30 min until a dark red solution was obtained. After obtaining the AuNSs, the pH was adjusted to 7–8 using a diluted NaOH solution [20].

Gold NRs (AuNRs) were synthesized using the modified seed-mediated growth method [25]. The seed was prepared by mixing 85 µL of a solution of HAuCl_4_ (29.4 mM) with 4.915 mL of Cetyl Trimethyl Ammonium Bromide (CTAB) (0.1 8 M) as a stabilizing agent and stirred for 5 min; then, 300 µL of a cold solution of NaBH_4_/NaOH (10 mM/0.01 M) was added to reduce the gold and was left stirring for 30 min. Subsequently, the growth solution was prepared by adding 170 µL of HAuCl_4_ gold solution (29.4 mM) to 9.83 mL of CTAB (0.1 M) and stirred for 10 min. Then, 1 mL of AgNO_3_ (10 mM) was added to the mixture and stirred for 30 seg. After time passed, 500 µL of hydroquinone (100 mM) was added, and it was left stirring until a change of color (from yellow to colorless) was noted. Finally, 160 µL of the seed was mixed with the growth solution and left to rest overnight [26].

### 3.3. Plasmon Resonance Assessment

The plasmon resonance of the AuNSs and AuNRs was assessed through UV spectroscopy, employing an Agilent 8453 UV-DAD spectrophotometer (Agilent Technologies, Santa Clara, CA, USA) controlled by Chemstation v.B04-02 software. The measurement configuration encompassed a resolution of 1 nm and a range from 190 to 1100 nm [26].

### 3.4. Determination of the Average Hydrodynamic Diameter and Polydispersity Index

The acquired nanosuspensions underwent characterization for their hydrodynamic diameter (size) and polydispersion index (PDI) using dynamic light scattering (DLS) technique with noninvasive dispersion, measured at an angle of 173° through a Zetasizer^®^ Nano ZS 90 instrument (Malvern Instruments, Malvern, UK). The refractive index used was 1.330. Before the measurements (conducted in triplicate), the samples were appropriately diluted (1:100) [5,24,26].

### 3.5. Nanoparticle Tracking Analysis NTA

The AuNS and AuNR concentrations were quantified through nanoparticle tracking analysis (NTA) employing a NanoSight NS300 system (NanoSight, Amesbury, UK). Before characterization, dilution of 1:100 was carried out using Milli-Q water [24].

### 3.6. Scanning Transmission Electron Microscopy (STEM)

To analyze the structure of the AuNSs and AuNRs within a liquid environment, the samples underwent observation through electron microscopy utilizing a scanning transmission electron microscope, the FEI Inspect F50. To achieve this, 20 µL of each sample was placed onto a Formvar/Carbon-Coated Copper 300 mesh grid for 2 min. Following this, the grid underwent a dual rinse with Milli-Q water, each lasting a minute, after which, it was carefully dried using absorbent Whatman paper [24,26].

### 3.7. Design of Matrices for 3D Printing

**Figure 17:**
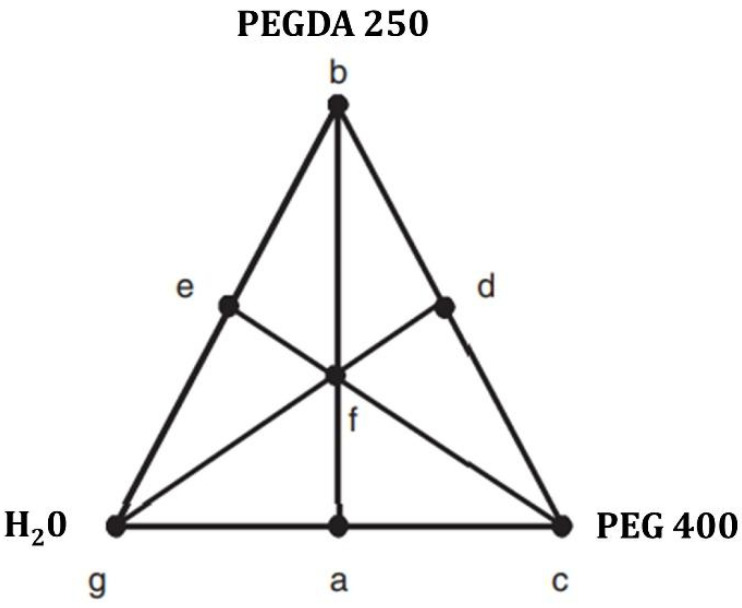
Mixture-simplex centroid design applied to the software Design Expert version 7.0.0.

For the manufacture of polymeric matrices as materials for 3D printing through photopolymerization in a vat, the solubility of the model drug niclosamide in ternary mixtures was studied. The proportions for preparing these mixtures were obtained using Design Expert software. The ternary experimental design was a mixed-center design. Different proportions of a photopolymer (PEGDA 250), a cosolvent (PEG 400), and water, in which the nanosystems were suspended, were combined in these mixtures (Figure 17). Solvent combinations were made, resulting in 13 different matrices, and the solubility of the drug was evaluated in each of them. The determinations were performed in triplicate [24].

### 3.8. Rheology

A rotational rheological test was conducted using a 50 mm diameter cone–plate system and an Anton Paar Physica MCR 301 instrument with a 10–5 mNm lower detection limit. To obtain information over a wide range of shear rates, the measurement was performed between 0.001 and 1000 s^−1^ [24].

### 3.9. The 3D-Printing Process

Cylinders of 3 × 5 mm were designed using Fusion360 software v.2.0 (Autodesk Inc., San Francisco, CA, USA) and then the print files were generated using Chitubox (CTB SDK, ShenZhen, China). A radiation time of 65 s was used for the first layer and 4 s for subsequent layers. The layer height was 0.05 mm, the lift distance was 6 mm, and the lift and retract speed was 180 mm/min. The prints were produced using an Anycubic Photon S printer (Anycubic, Shenzhen, China) and the printed objects were washed with Milli-Q water and cured at 405 nm for 1 min in an Anycubic Wash and Cure device (Anycubic, Shenzhen, China) [19,24].

### 3.10. Morphological Analysis

The samples were placed on a calibrated glass slide and observed under an optical microscope (OLYMPUS Model BX-51, OLYMPUS, Tokyo, Japan) at 10x magnification [24].

### 3.11. Fourier-Transform Infrared Evaluations

Fourier-transform infrared spectra (FTIR-ATR) of liquid and powder samples were obtained using an Agilent Technologies Cary 630 spectrophotometer. The samples were scanned from 4000 to 400 cm⁻^1^, with a resolution of 1 cm⁻^1^. The data analysis was conducted using OMNIC^®^ software (Thermo Fisher Scientific, St. Bend, OR, USA). The determinations were performed in triplicate [24,26].

### 3.12. Weight Uniformity

Following the guidelines indicated in United States Pharmacopeia (USP, 2007), the uniformity of the obtained forms’ weights was evaluated. For this, it was necessary to weigh ten equal-sized printlets, calculate the average mass, and verify that no more than two individual masses deviated by more than 5% from the average mass. Ten copies of each matrix were transported to the analytical balance to ascertain the weight of each print. These prints were meticulously weighed within a 1.5 mL Eppendorf tube. Before the weighing process, the balance was calibrated using the empty Eppendorf tube to ensure that only the weight of the print itself was recorded [19,24].

### 3.13. Scanning Electron Microscopy

Scanning electron microscopy (SEM) (FEI Inspect 50-USA with an accelerating voltage of 3–20 kV) at magnifications between 500x and 14,000x was used to analyze the surface morphology and the internal structure of the printlets was sputtered with Au before examination [24,26].

### 3.14. Evaluation of the Photothermal Effect

A 532 nm laser (LDCU5/9020, Power Technology, Alexander, AR, USA) and a 1064 nm laser (IQ1A350, Laseray Inc., Cleveland, OH, USA) were used to irradiate the AuNSs and AuNRs in a closed, dark chamber to assess the photothermal effect of the nanoparticles in the printed devices. The laser power used was 350 mW. Images were captured with a FLIR E8xt thermal camera (FLIR Systems Inc., Washington, DC, USA). Printed objects with AuNPs and control prints without nanoparticles were placed in 1.5 mL Eppendorf tubes with phosphate-buffered saline (PBS). Afterward, they were irradiated for 5 min, and the temperature changes were evaluated to determine the photothermal effect of the nanoparticles in the 3D prints [26].

### 3.15. Differential Scanning Calorimetry

Differential Scanning Calorimetry (DSC) tests were conducted to assess the thermal properties of the samples (approximately 5 mg). The samples were weighed and sealed in perforated aluminum pans and DSC measurements were performed using a Discovery DSC 25P instrument (TA Instruments, New Castle, DE, USA) under a dynamic atmosphere of N2 (50 mL/min) with a heating rate of 5 °C/min in a temperature range from 25 °C to 250 °C. The DSC cell was calibrated using indium (MP = 156.6 °C; Delta H fus = 28.54 J/g) [5,24].

### 3.16. Thermogravimetric Analysis

A Discovery HP TGA (TA Instruments, New Castle, DE, USA) was used for thermogravimetric analysis. Thermogravimetric analysis (TGA) curves were obtained at 25–350 °C under a dynamic atmosphere of N2 (50 mL/min) with a heating rate of 5 °C/min. The TRIOS program (USA) was employed to analyze the data [5,24].

### 3.17. Evaluation of Release Without Irradiation: Phase Solubility Equilibrium

The printed devices were placed in 1.5 mL Eppendorf tubes with PBS and subjected to continuous agitation at 250 rpm in a thermoshaker (37 °C). A 1 mL sample was taken for measurement in a UV–Vis spectrophotometer at 30 min, 60 min, 90 min, 120 min, and 4320 min (equivalent to 3 days). The sample taken for measurement was returned to the same tube to ensure that the concentration of released Niclosamide remained cumulative. The determinations were performed in triplicate [5,19].

### 3.18. Assessment of Drug Release by Laser Irradiation

To assess release through irradiation, the devices were placed in 1.5 mL Eppendorf tubes and irradiated for 15 min using a 532 nm laser (LDCU5/9020, Power Technology, Alexander, AR, USA) or a 1064 nm laser (IQ1A350, Laseray Inc., Cleveland, OH, USA), with the release of the model drug being recorded by measuring the UV–Vis spectrophotometry at 333 nm every one minute. The determinations were performed in triplicate [26].

### 3.19. Statistical Analysis

Each result was expressed with its mean value ± standard error (SD). GraphPad Prism 7.0 software (GraphPad Software, San Diego, CA, USA) was used for statistical analysis. An analysis of variance test was used for statistical comparison/analysis. A *p*-value of 0.05 or less was considered statistically significant [5,24].

## 4. Conclusions and Prospects

The potential of 3D printing in photothermal therapy presents significant promise. This technology facilitates the fabrication of highly customized medical devices tailored to individual patient needs, particularly in cancer treatment, where tumor characteristics can vary substantially. Moreover, 3D printing offers the capability to design intricate and microscopic structures that can enhance the effectiveness of photothermal therapy. It enables the creation of nanoparticles and nanosensors for controlled drug release and the monitoring of tissue response to treatment. These innovations can potentially revolutionize medicine, offering substantial improvements in patient outcomes.

In this study, we designed two novel 3D-printing materials for VAT photopolymerization by employing ternary mixtures of PEGDA 250/PEG400/H_2_O loaded with either AuNSs or AuNRs to achieve selective drug release through irradiation with different wavelengths of lasers (532 and 1064 nm). The process encompassed the synthesis of nanosystems, followed by verifying their colloidal stability and morphology. Subsequently, a mixed-centered design was employed to determine the combination of materials that yielded the highest solubility of the model drug. Based on this design, three matrices were selected, achieving acceptable Niclosamide solubility while exhibiting variable photopolymer concentrations to evaluate printability.

The 3D printing revealed a higher optical density of materials developed with Matrix 6 (90% PEGDA 250), greater geometric fidelity compared to the digital file, and increased weight uniformity. In-depth characterization of the printed materials confirmed the successful incorporation of nanosystems by demonstrating the photothermal effect. Analysis through DSC and TGA showed no degradation due to the irradiation process. Additionally, it was determined that the materials did not release the active compound without irradiation. Subsequent assessment of selective drug release through laser irradiation underscored the importance of nanoparticle concentration in enhancing the release rate.

In conclusion, from the designed matrices, it can be deduced that a suitable region was identified where the active compound was solubilized effectively, the materials were printable using the VAT photopolymerization technique, and the control of overactive compound release from the devices was achieved. For future work, it is imperative to precisely define the boundaries of this region to develop viable matrices for printing, potentially using other photopolymers such as PEGDA 575 and PEGDA 750. Future challenges will involve establishing a robust platform for loading multiple drugs and nanosystems to cover different irradiation ranges. More outstanding releases for less potent drugs will sometimes be necessary. Furthermore, considering materials’ cytotoxicity is crucial to ensure that the designed device is suitable for safe and effective pharmaceutical administration.

## Figures and Tables

**Table 1:** Characterization of AuNSs and AuNRs by Zeta potential and hydrodynamic diameter.

Nanosystem	Z Potential (mV) *	Hydrodynamic Diameter (nm) *	Hd. Transverse (nm) *	Hd. Longitudinal (nm) *	PDI *
AuNSs	−48 ± 2.8	27 ± 1.2			0.24
AuNRs	32 ± 0.3		4 ± 0.5	60 ± 16	0.47

* The determinations were performed in triplicate.

**Table 2:** The diverse array of component ratios involving PEGDA 250, PEG 400, and water of the 13 matrices under examination. The right-hand side of the table provides the 17 corresponding solubilities of Niclosamide in micrograms per milliliter (μg/mL) within each of these matrices. The selected matrices are highlighted in bold.

Run	A:PEGDA 250	B:PEG 400	C:H_2_0	NICLO SOLUBILITY (ug/mL)
1	0.2	0.35	0.45	54.4
**2**	**0.2**	**0.7**	**0.1**	**11,778.6**
3	0.316667	0.466667	0.216667	4773.3
4	0.55	0	0.45	218.4
**5**	**0.55**	**0.35**	**0.1**	**5370.7**
**6**	**0.9**	**0**	**0.1**	**4069.5**
7	0.666667	0.116667	0.216667	57.6
8	0.2	0	0.8	142.7
9	0.2	0	0.8	13.1
10	0.9	0	0.1	3974.7
11	0.433333	0.233333	0.333333	56.1
12	0.316667	0.116667	0.566667	16.0
13	0.2	0.7	0.1	13,395.8

**Table 3:** Selected formulation composition and drug loading (DL%).

Matrix	PEGDA 250 (%)	PEG 400 (%)	H_2_O (%)	NICLO (µg/mL)	DL%	AuNSs/AuNRs [pM]
2	0.2	0.7	0.1	11,700.0	1.06	17.6/2.66
5	0.55	0.35	0.1	5300.0	0.48	
6	0.9	0	0.1	4000.0	0.36	

**Table 4:** Weight distribution, niclosamide content per device, drug loading (DL%), and entrapment efficiency (EE%) in printed objects using Matrices 2, 5, and 6.

Matrix	Average Weight (mg)	Standard Deviation	Amount of Niclosamide per Device (mg)	DL%	EE%
2	1.97	0.34	0.02	0.96	90.67
5	2.05	0.29	0.01	0.45	95.02
6	14.97	0.11	0.05	0.33	91.85

## Data Availability

The data presented in this study are available on request from the corresponding author.
